# The Staphylococcus aureus Transcriptome during Cystic Fibrosis Lung Infection

**DOI:** 10.1128/mBio.02774-19

**Published:** 2019-11-19

**Authors:** Carolyn B. Ibberson, Marvin Whiteley

**Affiliations:** a School of Biological Sciences, Georgia Institute of Technology, Atlanta, Georgia, USA; b Emory-Children’s Cystic Fibrosis Center, Atlanta, Georgia, USA; c Center for Microbial Dynamics and Infection, Georgia Institute of Technology, Atlanta, Georgia, USA; University of Texas Southwestern Medical Center Dallas

**Keywords:** *Staphylococcus aureus*, RNA-seq, transcriptomics, machine learning, virulence, cystic fibrosis, human infection, virulence factors

## Abstract

Laboratory models have been invaluable for the field of microbiology for over 100 years and have provided key insights into core aspects of bacterial physiology such as regulation and metabolism. However, it is important to identify the extent to which these models recapitulate bacterial physiology within a human infection environment. Here, we performed transcriptomics (RNA-seq), focusing on the physiology of the prominent pathogen Staphylococcus aureus *in situ* in human cystic fibrosis (CF) infection. Through principal-component and hierarchal clustering analyses, we found remarkable conservation in S. aureus gene expression in the CF lung despite differences in the patient clinic, clinical status, age, and therapeutic regimen. We used a machine learning approach to identify an S. aureus transcriptomic signature of 32 genes that can reliably distinguish between S. aureus transcriptomes in the CF lung and *in vitro*. The majority of these genes were involved in virulence and metabolism and were used to improve a common CF infection model. Collectively, these results advance our knowledge of S. aureus physiology during human CF lung infection and demonstrate how *in vitro* models can be improved to better capture bacterial physiology in infection.

## Abstract (precis)

Although bacteria have been studied in infection for over 100 years, the majority of these studies have utilized laboratory and animal models that often have unknown relevance to the human infections they are meant to represent. A primary challenge has been to assess bacterial physiology in the human host. To address this challenge, we performed transcriptomics of S. aureus during human cystic fibrosis (CF) lung infection. Using a machine learning framework, we defined a “human CF lung transcriptome signature” that primarily included genes involved in metabolism and virulence. In addition, we were able to apply our findings to improve an *in vitro* model of CF infection. Understanding bacterial gene expression within human infection is a critical step toward the development of improved laboratory models and new therapeutics.

## INTRODUCTION

Staphylococcus aureus was first observed in pus from a surgical abscess in 1880 by surgeon Sir Alexander Ogston (1–3). Soon after its discovery, it was quickly appreciated that S. aureus was a formidable pathogen (4–8), and it is still a leading cause of bacterial infection worldwide. This versatile pathogen is well adapted to mammalian hosts (4, 5, 9–11). S. aureus is able to infect nearly every tissue in the body (4, 6, 7, 10), ranging from mild skin and soft tissue infections to potentially fatal and/or chronic conditions such as sepsis, osteomyelitis, endocarditis, and cystic fibrosis (CF) lung infection (4–8). In addition, S. aureus is a frequent human commensal, with ∼30% of the human population persistently colonized in the nasal cavity (12, 13).

Studies of S. aureus have centered on understanding mechanisms of virulence, regulation, and physiology and have typically been performed in liquid culture in a test tube or in animal infection models. These studies have collectively uncovered complex regulatory networks that integrate quorum sensing, two-component systems, and sensing of both internal (e.g., metabolite levels) and external (e.g., host substrates) cues (14–21). In addition, an impressive arsenal of >50 virulence factors and immune evasion strategies has been described (5–7, 10, 22).

While *in vitro* and animal models collectively have provided insights into the ways that S. aureus interacts with eukaryotic hosts and defined core aspects of S. aureus metabolism and regulation, it is often not clear how well and in what ways model systems replicate the physiology that occurs in human infection. For obvious ethical reasons, infections in humans are difficult to study *in situ*. However, advances in -omics techniques now allow for global assessments of gene transcription, protein levels, and metabolite production by bacteria in their native environments (23–27).

CF is a recessive genetic disease caused by mutations in the gene encoding the cystic fibrosis transmembrane conductance regulator, an ion channel that conducts chloride and bicarbonate across epithelial cell membranes, resulting in the accumulation of viscous mucus in the airways. Bacteria use this thick mucus as a growth environment, and CF patients experience frequent lung infections that begin in early childhood and persist throughout their life. These infections are the primary cause of morbidity and mortality in individuals with CF (28). S. aureus is the most common microbe isolated from expectorated CF patient sputum (29), and therefore, CF is a relevant infection for studying S. aureus physiology *in situ*.

Here, we use transcriptomics (RNA-seq) to assess S. aureus physiology during human CF lung infection. Building on a machine learning approach that was previously developed to study Pseudomonas aeruginosa human infections (24), we identified a transcriptomic signature of S. aureus during human CF lung infection. We defined a set of 32 genes, many of which are involved in virulence and metabolism, that are sufficient to distinguish between transcriptomes from human CF lung infection and *in vitro* transcriptomes. We further showed how these data can help improve laboratory models to better mimic human infection by adding a host molecule to a CF *in vitro* model, which altered the expression of virulence genes and increased the similarity of the S. aureus transcriptome in that model to that in CF sputum. Our ultimate goal is to provide benchmark data on S. aureus transcription *in situ* and to develop a framework for assessing bacterial physiology within human infection.

## RESULTS

### Transcriptomes used in this study.

In this work, we performed RNA-seq on S. aureus from both human clinical samples and *in vitro* cultures. The human clinical samples are primarily from expectorated CF sputum and are the focus of this paper. CF sputum samples were collected from the Emory Cystic Fibrosis Center (*n* = 9) or from Denmark (*n* = 1) from adult patients who were classified as clinically stable (Table 1). We did not target a particular cohort of patients, other than CF patients who can expectorate sputum, as our goal was to define the core ways that S. aureus human transcriptomes differ from *in vitro* transcriptomes. One limitation of clinical samples is that the strains that comprise the reads in our samples are unknown; however, we can make broad classifications, such as whether the S. aureus strains are methicillin sensitive (MSSA) or methicillin resistant (MRSA). To do this, we determined if the strains in our samples were MRSA or MSSA by assessing how many S. aureus reads mapped to *mecA*. We found that 8/10 sputum samples were likely dominated by MRSA (Table 1) with >10 reads mapping to *mecA* (mean read count per gene in the sputum samples was 66 ± 20, standard error of the mean).

**TABLE 1:** CF sputum samples used in this study

Human sputum sample	Source	Total no. of reads	No. of reads mapped to:		MSSA or MRSA^*a*^	Recent sputum microbiology^*b*^
			S. aureus	*mecA*		
EM3	GA, USA	54,478,195	2,569,031	4,674	MRSA	MSSA, *P. aeruginosa*
EM13	GA, USA	77,035,116	990,342	474	MRSA	P. aeruginosa
EM15	GA, USA	110,756,562	622,666	15	Likely MRSA	*Achromobacter*, MSSA
EM18	GA, USA	50,839,416	456,406	38	Likely MRSA	*Achromobacter*, MSSA
EM22	GA, USA	29,554,488	405,887	6	MSSA	MSSA
EM47	GA, USA	49,914,107	1,288,259	128	MRSA	MSSA
EM48	GA, USA	56,958,617	1,239,803	28	Likely MRSA	MSSA, *P. aeruginosa*
EM58	GA, USA	123,479,263	960,385	22	Likely MRSA	*Burkholderia cepacia*, MRSA, P. aeruginosa
EM61	GA, USA	78,248,618	193,333	5	MSSA	MSSA, *P. aeruginosa*
G	Denmark	78,869,961	624,172	48	Likely MRSA	P. aeruginosa, S. aureus

a MRSA and MSSA designations were determined by the number of S. aureus reads mapping to *mecA* in a pangenome of 12 S. aureus strains. Strains were designated “likely MRSA” if >10 reads mapped to *mecA* and MRSA if >100 reads mapped.

b Recent sputum microbiology is the associated clinical microbiology culture metadata for the sample.

In addition to the CF sputum transcriptomes that we collected, we also included two non-CF transcriptomes from human S. aureus infections, one from a previously published joint infection (30) and the other from a chronic wound; these additional samples allow us to make comparisons for human infections outside the CF lung. Our *in vitro* data are composed of 22 RNA-seq data sets from our and other laboratories (30–32) during growth under a variety of conditions, including rich complex medium (tryptic soy broth [TSB], lysogeny broth [LB], brain heart infusion broth [BHI]) and chemically defined medium with either glucose or amino acids as a primary carbon and energy source. In addition, a number of different S. aureus isolates were used in the *in vitro* studies, including the USA300 community-associated methicillin-resistant strain LAC* (33), the closely related USA300 strains JE2 (34) and UAMS-1790 (35), the USA200 strain UAMS-1 (17), the laboratory strain SH1000 (36), and the clinical isolate SAU060112 (30). A complete list of the *in vitro* samples used in this study is included in Table 2.

**TABLE 2:** *In vitro* samples used in this study

Sample	Growth medium	Growth phase	Strain	Source or reference
Samples used for all assays				
1	CDM + 20 mM glucose	Mid-logarithmic	LAC*	This study
2	CDM + 20 mM glucose	Mid-logarithmic	LAC*	This study
3	CDM + 20 mM glucose	Early stationary	LAC*	This study
4	CDM + 20 mM glucose	Early stationary	LAC*	This study
5	CDM + 20 mM glucose	Early stationary	LAC*	This study
6	CDM + no additional carbon	Mid-logarithmic	LAC*	This study
7	CDM + no additional carbon	Mid-logarithmic	LAC*	This study
8	CDM + no additional carbon	Early stationary	LAC*	This study
9	CDM + no additional carbon	Early stationary	LAC*	This study
10	BHI	Mid-logarithmic	LAC*	This study
11	BHI	Mid-logarithmic	LAC*	This study
12	BHI	Mid-logarithmic	LAC*	This study
13	BHI	Mid-logarithmic	LAC*	This study
14	LB	Mid-logarithmic	SAU060112	30
15	LB	Mid-logarithmic	SAU060112	30
16	LB	Mid-logarithmic	SAU060112	30
17	TSB	Early stationary	JE2	32
18	TSB	Early stationary	JE2	32
19	TSB	Early stationary	JE2	32
20	TSB	Early stationary	UAMS-1790	31
21	TSB	Mid-logarithmic	SH1000	31
22	TSB	Mid-logarithmic	UAMS-1	31
Samples used for only machine learning and model improvement analyses				
23	SCFM2	Mid-logarithmic	LAC*	This study
24	SCFM2	Mid-logarithmic	LAC*	This study
25	SCFM2	Mid-logarithmic	LAC*	This study
26	SCFM2	Mid-logarithmic	LAC*	This study
27	SCFM2	Late logarithmic	LAC*	This study
28	SCFM2	Late logarithmic	LAC*	This study
29	SCFM2	Late logarithmic	LAC*	This study
30	SCFM2	Late logarithmic	LAC*	This study
31	SCFM2	Late logarithmic	LAC*	This study
32	SCFM2 + HNP-1	Mid-logarithmic	LAC*	This study
33	SCFM2 + HNP-1	Mid-logarithmic	LAC*	This study

### The S. aureus human CF transcriptome is distinct from *in vitro* models.

**FIG 1:**
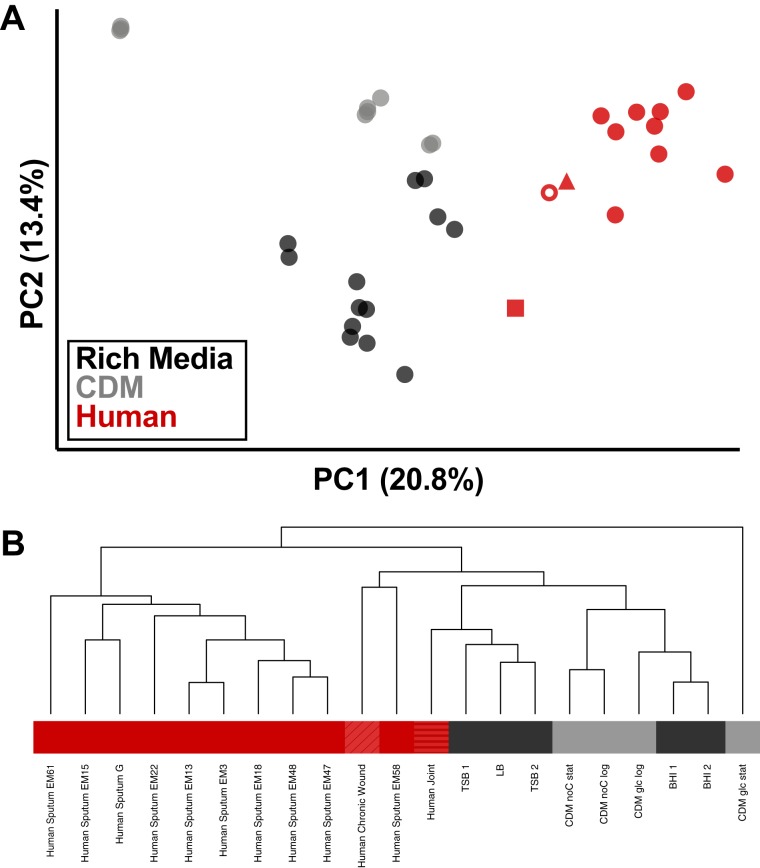
S. aureus transcriptomes from human infection cluster independently from *in vitro* samples. (A) Principal-component analysis of variance stabilizing transformation (VST)-normalized reads from S. aureus RNA-seq. Transcriptomes from human infection are shown in red, with closed circles representing human CF sputum from Emory (*n* = 9), open circle representing human CF sputum from Denmark (*n* = 1), square representing human joint infection (*n* = 1), and triangle representing human chronic wound infection (*n* = 1). Transcriptomes from *in vitro* conditions are shown indicating the type of medium in which they were grown, with black representing rich medium (LB, TSB, or BHI; *n* = 13) and gray representing chemically defined medium with two separate carbon sources (CDM; *n* = 9). (B) Hierarchal clustering of sample types using normalized reads per gene for each condition. Under conditions with replicates (*in vitro*), replicates were averaged and the mean counts for each gene were used. Black indicates samples from rich medium, gray indicates samples from CDM, and red indicates samples from human infection (solid, sputum; diagonally striped, chronic wound; horizontally striped, joint). For *in vitro* samples, “CDM glc log” is samples 1 and 2, “CDM glc stat” is samples 3 to 5, “CDM noC log” is samples 6 and 7, “CDM noC stat” is samples 8 and 9, “BHI 1” is samples 10 and 11, “BHI 2” is samples 12 and 13, “LB” is samples 14 to 16, “TSB 1” is samples 17 to 19, and “TSB 2” is samples 20 to 22 listed in Table 2.

When assessing gene expression in clinical samples, one concern is biasing the results due to the presence/absence of genes resulting from differences in strain background when making comparisons between conditions. To address this, we constructed a reduced gene set consisting of only 1,960 genes (∼70% of the total genes in an S. aureus genome) that are conserved across a set of 15 genetically diverse S. aureus strain backgrounds (see Table S1 in the supplemental material) and mapped transcriptomes to this reduced gene set (9). In addition, we removed reads mapping to tRNAs and rRNAs from our data sets, since any potential differences in rRNA depletion during library preparation can affect normalization and fold change calculations. A principal-component analysis (PCA) based on this resulting data set shows that the human CF sputum samples cluster distinctly from our *in vitro* models (Fig. 1A and Fig. S1A), and they cluster remarkably closely across all 10 patients despite differences in coinfecting microbes, patient status, therapeutic regimen, and geographic location of the clinic. In fact, S. aureus sputum transcriptomes from different patients are more closely associated on the PCA than *in vitro* samples during different growth phases (Fig. 1A and Fig. S1A). In addition to the CF sputum samples, we also included S. aureus transcriptomes from 1 human joint infection (30) and 1 human chronic wound infection in this analysis to determine if S. aureus transcription in human infection in general was similar. We found these samples clustered with the CF sputum samples in our PCA (Fig. S1A), indicating similarities in gene expression that may be shared across different infection types. Additional principal components (PC) are shown in Fig. S2 as well as a scree plot of the first 20 PCs. We have also included a PCA using only those genes that have reads mapping to them in all of our clinical samples (1,046 common genes) to control for the potential presence/absence of genes in the samples impacting the clustering (Fig. S3). We find similar patterns of clustering with this methodology. In addition to principal-component analysis, we assessed the similarity of S. aureus gene expression across our sample types with hierarchal clustering (Fig. 1B). Through this analysis, we found that the human CF sputum samples cluster independently from the *in vitro* model systems, confirming the PCA results. Interestingly, while we find that the human wound sample clusters with the CF sputum, the human joint infection sample clusters with the *in vitro* samples by this measure.

10.1128/mBio.02774-19.1 FIG S1 *k*-means analysis of the principal-component analysis of S. aureus RNA-seq from 1,960 conserved genes. (A) *k*-means clusters of first two principal components from Fig. 1. Cluster 1 is shown in red, and cluster 2 is shown in black. Asterisks indicate the centers of the clusters. (B) Euclidean distances of the PCA points to the respective centers. The bar indicates the mean, and error bars show 95% confidence intervals. Download FIG S1, EPS file, 0.8 MB. Copyright © 2019 Ibberson and Whiteley. 2019 Ibberson and Whiteley This content is distributed under the terms of the Creative Commons Attribution 4.0 International license.

10.1128/mBio.02774-19.2 FIG S2 Principal-component analysis of VST-normalized read counts of S. aureus RNA-seq data of 1,960 genes conserved in 15 diverse strains under a variety of conditions. (A) Scree plot showing the variance represented by the first 20 principal components. (B to D) Plots of other combinations of principal components: principal component 2 versus 3 (B), principal component 1 versus 3 (C), and principal component 1 versus 4 (D). Transcriptomes from human infection are shown in red with circles representing human CF sputum (*n* = 10), squares representing human joint infection (*n* = 1), and triangles representing human chronic wound infection (*n* = 1). Transcriptomes from *in vitro* conditions are shown indicating the growth medium, with black representing rich medium (LB, TSB, or BHI; *n* = 13) and gray representing chemically defined medium with two carbon sources (CDM; *n* = 9). Download FIG S2, EPS file, 0.2 MB. Copyright © 2019 Ibberson and Whiteley. 2019 Ibberson and Whiteley This content is distributed under the terms of the Creative Commons Attribution 4.0 International license.

10.1128/mBio.02774-19.3 FIG S3 Principal-component analysis of VST-normalized read counts of S. aureus RNA-seq data from 1,046 genes that had at least 1 read in all clinical samples under a variety of conditions. (A) Principal-component analysis of VST-normalized reads from S. aureus RNA-seq. Transcriptomes from human infection are shown in red, with circles representing human CF sputum (*n* = 10), squares representing human joint infection (*n* = 1), and triangles representing human chronic wound (*n* = 1). Transcriptomes from *in vitro* conditions are shown indicating the growth medium, with black representing rich medium (LB, TSB, or BHI; *n* = 13) and gray representing chemically defined medium with two carbon sources (CDM; *n* = 9). (B) Scree plot showing the variance represented by the first 20 principal components. (C) Plot of the first 10 principal components. Transcriptomes from human infection are shown in red, with circles representing human CF sputum (*n* = 10), squares representing human joint infection (*n* = 1), and triangles representing human chronic wound (*n* = 1). Transcriptomes from *in vitro* conditions are shown indicating the type of medium in which they were grown, with black representing rich medium (LB, TSB, or BHI; *n* = 13) and gray representing chemically defined medium with two carbon sources (CDM; *n* = 9). Download FIG S3, EPS file, 0.2 MB. Copyright © 2019 Ibberson and Whiteley. 2019 Ibberson and Whiteley This content is distributed under the terms of the Creative Commons Attribution 4.0 International license.

10.1128/mBio.02774-19.5 TABLE S1 The 15 diverse S. aureus strains used to construct the reduced gene set of 1,960 conserved genes. Download Table S1, DOCX file, 0.01 MB. Copyright © 2019 Ibberson and Whiteley. 2019 Ibberson and Whiteley This content is distributed under the terms of the Creative Commons Attribution 4.0 International license.

### Expression of virulence factors and metabolic genes differentiate *in vivo* and *in vitro* samples.

Since the human and *in vitro* samples clustered independently, we were next interested in determining which functional categories differed between these sample types. We performed differential expression analysis with DESeq2 (37) comparing our *in vitro* conditions collectively to S. aureus expression in CF sputum to make broad assessments about *in vitro* versus *in vivo* growth. We focused on those genes with the largest changes between the two groups to make robust observations (adjusted *P* value of <0.05, >4-fold change [Data Set S1]), and 271 genes were differentially expressed by this measure. To determine functional relationships, genes were annotated with TIGRFAM categories (38). However, as the TIGRFAM categories did not include virulence factors, annotations for these genes were curated from the literature (6–8, 18, 22, 39–41). Some of the most differentially expressed genes in humans compared to *in vitro* were virulence factors, the majority of which had increased expression in CF sputum, and included gamma-hemolysin (*hlgABC*), superantigen-like proteins (*ssl1*, *ssl2*, *ssl3*, *ssl5*, *ssl9*, *ssl10*, *ssl12*, *ssl13*, and *ssl14*), leukocidins (*lukG*), extracellular matrix binding proteins (*emp*, *scc*, and *fnbA*), and exopolysaccharide (*icaABC*). In fact, virulence factors as a category were enriched (Fisher’s exact test, adjusted *P* value of <0.05) in the differentially expressed genes in CF sputum samples compared to *in vitro*. Normalized read counts for all S. aureus virulence factors conserved across the 15 strain backgrounds are shown in Fig. S4.

10.1128/mBio.02774-19.4 FIG S4 Expression of conserved virulence factors *in vitro* and in human infection. Normalized read counts of S. aureus virulence factors that are conserved across 15 diverse strains. Black symbols are from *in vitro* conditions, and red symbols are from human samples. (A) Virulence factors localized in the cytoplasm; (B) secreted virulence factors; (C) surface-associated virulence factors. Download FIG S4, EPS file, 0.6 MB. Copyright © 2019 Ibberson and Whiteley. 2019 Ibberson and Whiteley This content is distributed under the terms of the Creative Commons Attribution 4.0 International license.

10.1128/mBio.02774-19.7 DATA SET S1 Differential gene expression using DESeq2 comparing S. aureus transcriptome in human sputum infection versus all *in vitro* conditions. Download Data Set S1, XLSX file, 0.3 MB. Copyright © 2019 Ibberson and Whiteley. 2019 Ibberson and Whiteley This content is distributed under the terms of the Creative Commons Attribution 4.0 International license.

**FIG 2:**
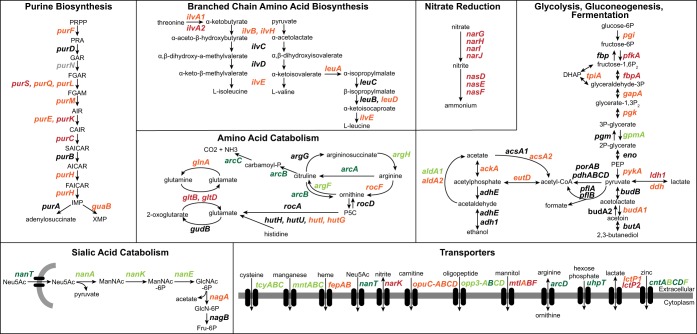
Metabolic pathways that are differentially expressed in human sputum compared to *in vitro* growth. A subset of notable metabolic pathways that showed differential expression (DESeq2, adjusted *P* value of <0.05, >2-fold change) in human sputum compared to exponential growth in chemically defined medium with glucose as a primary carbon source and/or compared to all *in vitro* conditions. Genes labeled in dark green indicate increased expression in CF sputum in both comparisons, light green indicates increased expression under one condition, orange indicates decreased expression under one condition, and red indicates decreased expression in sputum versus both *in vitro* comparisons. Pathways for purine biosynthesis and arginine biosynthesis were adapted from previous studies (58–60). Abbreviations for chemical compounds are in parentheses: *N*-acetylneuraminic acid (Neu5Ac), *N*-acetylmannosamine (ManNAc), *N*-acetylglucosamine-6-phosphate (GlcNAc-6P), glucosamine-6-phosphate (GlcN-6P), fructose-6-phosphate (Fru-6P), phosphoribosyl pyrophosphate (PRPP), 5′-phosphoribosylamine (PRA), glycine amide ribonucleotide (GAR), 5′-phosphoribosyl-*N*-formylglycineamide (FGAR), 5′-phophoribosylformylglycinamidine (FGAM), 5′-aminoimidazole ribotide (AIR), 5′-phosphoribosylaminoimidazolesuccinocarboxamide (SAICAR), 5′-aminoimidazole-4-carboxamide ribonucleotide (AICAR), 5′-formamidoimidazole-4-carboxamide ribotide (FAICAR), 5′phosphoribosyl-4-carboxy-5-aminoimidazole (CAIR).

In addition to virulence factors, many metabolic genes were differentially expressed *in vivo* compared to *in vitro* (adjusted *P* value of <0.05, >2-fold change [Data Set S1]). However, it can be challenging to infer host nutritional levels by comparison to S. aureus transcriptomes from complex conditions with unknown metabolite levels such as those found in rich culture media like BHI, TSB, and LB. Since many of our *in vitro* data sets were from these rich medium conditions, to better understand the *in vivo* nutritional environment, we wanted to also compare the human transcriptomes to a well-controlled *in vitro* condition under which the metabolite levels are known. We decided to compare the CF sputum transcriptomes to exponential growth in chemically defined medium (CDM) with glucose as a primary carbon source. We found that 183 genes were differentially expressed (adjusted *P* value of <0.05, >2-fold change in expression) between these conditions, and a complete list of these genes is included in Data Set S2. Some notable pathways that were differentially expressed are shown in Fig. 2 and include purine biosynthesis, amino acid catabolism, nitrate reduction, and transporters.

10.1128/mBio.02774-19.8 DATA SET S2 Differential gene expression using DESeq2 comparing S. aureus transcriptome in human sputum infection versus chemically defined medium with glucose as a carbon source. Download Data Set S2, XLSX file, 0.2 MB. Copyright © 2019 Ibberson and Whiteley. 2019 Ibberson and Whiteley This content is distributed under the terms of the Creative Commons Attribution 4.0 International license.

When taken together, these two comparisons (human sputum versus all *in vitro* and human sputum versus CDM glucose) allow us to make general insights into the nutritional status of S. aureus in the CF lung. (i) S. aureus is likely respiring oxygen and not fermenting. S. aureus gene expression under anaerobic conditions has been carefully measured through transcriptomics and proteomics (42). We find the transcriptomic metabolic profile of S. aureus in the CF lung is nearly opposite that of S. aureus grown anaerobically, with low expression of genes involved in fermentation and acquisition of the alternative electron acceptor nitrate, even compared to aerobic conditions (Fig. 2; Data Sets S1 and S2). Therefore, it is likely that oxygen is available and is being used by S. aureus. However, oxygen is likely relatively low as the high-affinity cytochrome *bd* oxidase (*cydAB*) has increased expression *in vivo* compared to *in vitro* growth (Fig. 2; Data Set S3 [these data have been derived and reformatted from Data Sets S1 and S2]). (ii) S. aureus is able to acquire iron in the CF lung, although its concentration is likely low. The biosynthetic pathway for the S. aureus siderophore staphyloferrin B had increased expression in human infection (Data Set S3), indicating that the CF lung is a low-iron environment where S. aureus is likely scavenging Fe^3+^. However, there was no change in expression for any of the Fe^2+^ transporters, and a number of heme uptake genes actually showed reduced expression in the CF lung (43). (iii) Other key metals such as manganese and zinc are likely limiting *in vivo* as, in general, transporters for these have increased expression in CF lung infection compared to *in vitro* (Fig. 2; Data Set S3). Additionally, *in vivo*, the expression of genes involved in the biosynthesis of the broad-spectrum metallophore staphylopine (44) are also increased.

10.1128/mBio.02774-19.9 DATA SET S3 Differential expression of metabolic genes from DESeq2 analyses. Download Data Set S3, XLSX file, 0.03 MB. Copyright © 2019 Ibberson and Whiteley. 2019 Ibberson and Whiteley This content is distributed under the terms of the Creative Commons Attribution 4.0 International license.

### Identification of a transcriptional signature of S. aureus in human CF infection.

**FIG 3:**
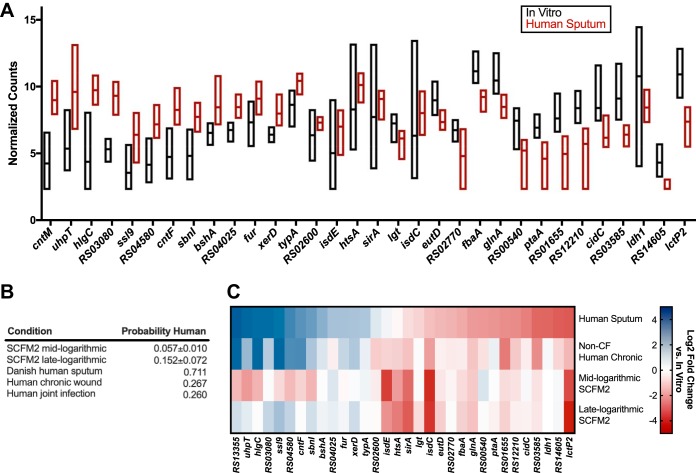
A subset of 32 genes is sufficient to distinguish S. aureus transcriptomes from human CF lung infection and those grown *in vitro*. (A) The *x* axis lists the 32 genes used to build our SVM model. VST-normalized read counts are plotted on the *y* axis. Black bars are samples grown *in vitro* (*n* = 22), and red bars are samples from human CF sputum (*n* = 9). Bars indicate the range of counts with a line that indicates the mean within the group. (B) Table showing the probability of the transcriptome from each condition to be from a human infection when classified by our SVM model. The standard deviation of the probability for either 4 replicates (mid-logarithmic) or 5 replicates (late logarithmic) is shown for the SCFM2 conditions. (C) Heat map showing the log_2_ fold change of S. aureus transcriptomes in human sputum, non-CF human chronic infection, mid-logarithmic growth in SCFM2, or late-logarithmic growth in SCFM2 compared to *in vitro* growth for the 32-gene transcriptomic signature used in the SVM model.

One goal of this work was to evaluate the ways in which model systems recapitulate human infection. While basic comparisons between our conditions are insightful, machine learning approaches can be more useful for probing differences between *in vitro* and human S. aureus transcriptomes. Therefore, we were next interested if we could determine with a machine learning approach a transcriptional signature that could differentiate between S. aureus CF sputum and *in vitro* transcriptomes. We trained our model with 9 human CF sputum samples from the Emory CF clinic and our 22 *in vitro* data sets. We used a filter wrapper in the mlr R package to select 50 genes that resulted in the largest information gain (*FSelectorRcpp_information.gain*) and best differentiated between data types in a support vector machine (SVM) model. We performed both 10-fold cross-validation and leave-one-out cross-validation of the SVM training process and feature selection using our test data set and had 100% accuracy with both methods. This identified 32 features (genes) that were used by the SVM to differentiate human and *in vitro* S. aureus transcriptomes and were conserved across all iterations of our validation methods (Fig. 3A). These genes are involved in a variety of functions for S. aureus, including metal acquisition (*sirA*, *sbnI*, *isdC*, *isdE*, *htsA*, and *cntF*), metabolism (*uhpT*, *bshA*, *htsA*, *eutD*, *fbaA*, *glnA*, *ptaA*, *cidC*, *ldh1*, and *lctP2*), and virulence (*hlgC* and *ssl9*). Figure 3A shows the normalized read counts for these genes. A key point is that these are not necessarily all of the important genes that can differentiate between the sample types, nor are they necessarily the most differentially expressed. However, they are effective in discriminating between human CF sputum and *in vitro* samples when used together. Of note, although some of these genes are coregulated (*ldh1* and *lctP2*; *fur*, *sbnI*, and *sirA*; and *isdE* and *isdC*), the performance of this gene set is not reliant on coregulated genes (Table S2).

10.1128/mBio.02774-19.6 TABLE S2 Assessment of SVM model performance after removing coregulated genes from the 32-gene transcriptional signature of human CF infection. Download Table S2, DOCX file, 0.01 MB. Copyright © 2019 Ibberson and Whiteley. 2019 Ibberson and Whiteley This content is distributed under the terms of the Creative Commons Attribution 4.0 International license.

### Using the SVM model to classify human and *in vitro* model transcriptomes.

In the SVM model, we used only the CF sputum transcriptomes obtained from Emory samples and did not include the human CF sputum sample from Denmark or the human joint and chronic wound infection samples. The rationale was that restriction to these transcriptomes would allow us to test the robustness of our predictive models on human transcriptomes from different clinics and different infection sites. We found the Denmark CF sputum sample was classified correctly as being a transcriptome from human CF sputum (Fig. 3B) using our model. This supports that our model is accurate in classifying S. aureus CF transcriptomes even from different countries and clinics. However, our model appears to be specific to human sputum as it classified the human joint and chronic wound infections as *in vitro* (Fig. 3B).

Model systems are often used to study human infections, including those in CF. Over the past decade, our laboratory has developed a synthetic sputum medium (SCFM2) that is meant to mimic the physical and chemical properties of CF sputum (45, 46). This model has been valuable for studying P. aeruginosa physiology in the CF lung but has not been evaluated in a comprehensive manner for studying S. aureus CF infection. Therefore, we used our SVM classification scheme to classify S. aureus SCFM2 transcriptomes as human sputum or *in vitro*. We found that transcriptomes from S. aureus grown in SCFM2 at two growth phases (mid- and late exponential) were classified as *in vitro* (Fig. 3B).

As the human samples outside the CF lung and SCFM2 were not classified as human CF sputum by our classification scheme, we were interested in the ways that these transcriptomes “failed” to be classified as CF sputum. It is important to note that the SVM classification scheme utilizes only the 32 genes that were identified as most discriminatory between CF sputum and *in vitro* transcriptomes. Thus, we next asked which of these 32 genes were not expressed similarly to CF sputum in the human infections outside the CF lung and in SCFM2, with the rationale that this comparison will provide insight into why these transcriptomes were classified as *in vitro*. To have increased statistical power, we treated the two non-CF human chronic infections as replicates. Figure 3C shows that while some genes in the non-CF human and SCFM2 transcriptomes have similar expression levels as our CF sputum transcriptomes (e.g., *isdC*, *sirA*, *lctP2*, and *glnA*), many genes involved in metabolism (*typA*, *bshA*, *htsA*, *eutD*, *fbaA*, *ptaA*, *cidC*, and *ldh1*) and iron acquisition (*isdE* and *sirA*) had a different expression profile than that in the CF sputum and were more similar to *in vitro* conditions. Additionally, the fold change of a number of genes for S. aureus grown in SCFM2 showed the opposite trend from the human sputum samples or was more similar to the *in vitro* value (Fig. 3C). A complete gene list containing the results of these comparisons is shown in Data Set S4.

10.1128/mBio.02774-19.10 DATA SET S4 Differential gene expression using DESeq2 comparing S. aureus gene expression in SCFM2 to that in human CF sputum. Download Data Set S4, XLSX file, 0.2 MB. Copyright © 2019 Ibberson and Whiteley. 2019 Ibberson and Whiteley This content is distributed under the terms of the Creative Commons Attribution 4.0 International license.

### Addition of human neutrophil peptide 1 improves the accuracy of SCFM2.

**FIG 4:**
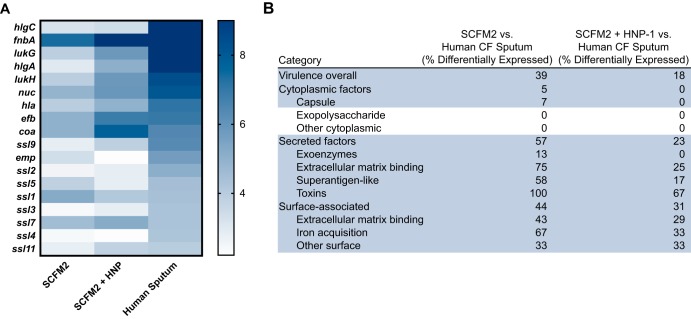
(A) Heat map showing the variance stabilizing transformation-normalized read counts of S. aureus in SCFM2 at mid-logarithmic phase or SCFM2 plus HNP-1 at mid-logarithmic phase or in human sputum for virulence factors known to be directly regulated by the SaeRS two-component system (47). Blue indicates higher read counts. (B) Differential gene expression of S. aureus virulence factors from transcriptomes grown in SCFM2 at mid-logarithmic phase or SCFM2 plus HNP-1 at mid-logarithmic phase compared to transcriptomes from human sputum. Categories that shifted with the addition of HNP-1 are highlighted in blue. The percent differentially expressed genes (>2-fold change in expression, adjusted *P* value of <0.05) in each category was calculated. SCFM2 experiments were performed at mid-logarithmic phase.

Can we use our gene expression comparisons and SVM approach to improve SCFM2 as a model of S. aureus CF? One of the most striking outcomes of our *in vitro*-CF sputum comparisons and the SVM was that genes encoding a number of virulence factors were significantly reduced during *in vitro* growth, including growth in SCFM2. Many of these virulence factors are controlled by the SaeRS system. SaeRS is a two-component regulatory system composed of a membrane-bound histidine kinase and cognate response regulator (47) and has been shown to be activated by components of the innate immune system, particularly neutrophils and a human antimicrobial alpha-defensin peptide (16). Upon infection, the most prominent cell type recruited to the CF lung is neutrophils. As neutrophils produce an alpha-defensin referred to as human neutrophil peptide (HNP-1), we hypothesized that addition of HNP-1 to SCFM2 might induce the SaeRS system and thus improve the ability of the model to mimic S. aureus transcription in CF sputum. To test this hypothesis, we added HNP-1 (16) to SCFM2 at a relevant physiological concentration and grew the samples to mid-exponential phase for transcriptomic analysis. The addition of HNP-1 to SCFM2 increased expression of secreted and surface-associated factors to levels more similar to those in the CF lung, reducing the number of differentially expressed genes in SCFM2 containing HNP-1 versus SCFM2 alone compared to human sputum (Fig. 4). Genes whose expression became more similar to that in the CF lung included *lukGH* (encoding a leukocidin), *nuc* (encoding nuclease), *efb* (encoding a fibrinogen binding protein), and *fnbA* (encoding fibronectin binding protein A), which have all been shown to be directly controlled by the SaeRS system (47) (Fig. 4).

## DISCUSSION

It is critical to study the physiology of organisms within their natural environment. However, until recently, studying bacterial pathogens within human infection has been technically prohibitive. Our goal was to expand the S. aureus human transcriptomic data from one sample with high sequencing coverage (30) as well as to provide benchmark data and a framework to assess S. aureus physiology *in situ* within human infection. To do this, we performed RNA-seq analysis on 10 human CF lung infection samples containing S. aureus and analyzed these samples in the context of 22 *in vitro* transcriptomes from a variety of conditions. We used RNA-seq data for this study as it is a quantitative, highly robust technique that can be performed with human-derived infection samples. In addition, mRNA levels can be used to infer S. aureus functions from clinical samples, thus providing mechanistic insights into the human growth environment. We found that the S. aureus CF sputum sample transcriptomes were highly similar to one another (Fig. 1), even more so than the *in vitro* transcriptomes (see Fig. S1 in the supplemental material), despite differences in clinic location, antibiotic regimen, and comorbidities (Fig. 1). This indicates that S. aureus has a definable functional repertoire during CF infection, suggesting that one can make informed *a priori* assumptions about the physiology of S. aureus in most CF lung infections.

We found that many metabolic pathways were differentially expressed in CF sputum than under the *in vitro* conditions. In general, biosynthetic pathways were reduced *in vivo*, while transporters and catabolic pathways often had increased expression (Fig. 2; Data Sets S1 and S2). Collectively, this indicates that the CF lung is nutrient rich; therefore, S. aureus does not need to synthesize a number of precursor metabolites. An interesting finding was that genes involved in fermentation were reduced in CF sputum (Fig. 2; Data Set S1). It is generally accepted that S. aureus is pushed toward a fermentative metabolism *in vivo* in CF sputum, particularly when in the presence of P. aeruginosa (48, 49). The clinical microbiology of our sputum samples indicates that 8 of these 10 patients harbor P. aeruginosa along with S. aureus in their lungs (Table 1); thus, it is intriguing that S. aureus exhibits reduced expression of fermentative pathways in the CF lung compared to monoculture planktonic growth in the lab. These data provide support for the hypothesis that P. aeruginosa and S. aureus do not interact by these *in vitro* observed mechanisms in the CF lung. One possible explanation is that although both of these species are present in the CF sputum samples, they could be spatially segregated, thus preventing sustained interactions (50), or that the number of these bacteria, which are not known in these samples, are not sufficient to support an interaction. Regardless, our approach provides benchmark human infection transcriptomic data to begin to approach such questions in complex environments such as CF lung infections.

Using a machine learning approach, we identified a subset of 32 genes that could reliably distinguish between CF sputum transcriptomes and those from *in vitro* (Table 3). While this transcriptomic signature of human CF lung infection could accurately classify a CF sputum sample from another clinic and country (Fig. 3B), it classified S. aureus transcriptomes from a human joint infection as well as a human chronic wound infection as more likely to be *in vitro* (Fig. 3B). The genes that most differentiated the non-CF human infections from those in CF sputum in this transcriptomic signature were involved in metabolism and iron acquisition (Fig. 3C). These data indicate that S. aureus has a distinct metabolic profile in different human infections, which is important to consider as the metabolic status of bacteria has been shown to impact the efficacy of antibiotics (51). Additionally, this demonstrates that there are easily distinguished gene expression patterns that can differentiate CF S. aureus lung infections from human infections at other sites. This could be due to the unique infection dynamics of the CF lung, in which microbes colonize the lung where they can evolve for years in the presence of therapeutic treatments. It should be pointed out that while one can discriminate between CF lung and non-CF lung in human infection transcriptomes, expression levels of many genes were highly similar, supporting that there are many S. aureus functional similarities during human infection. Thus, while we can discriminate among S. aureus human infection types using SVM, this does not imply that these conditions do not have functional similarities.

**TABLE 3:** Thirty-two genes that comprise a transcriptional signature that can reliably distinguish between S. aureus transcriptomes from *in vitro* growth and those from human CF sputum

Locus tag	Gene	Function^*a*^
SAUSA300_RS00540		AraC family transcriptional regulator
SAUSA300_RS00605	*sirA*	Iron ABC transporter substrate-binding protein
SAUSA300_RS00650	*sbnI*	Siderophore biosynthesis protein
SAUSA300_RS01135	*uhpT*	Hexose phosphate transporter
SAUSA300_RS01250	*ldh1*	l-Lactate dehydrogenase
SAUSA300_RS01655		PTS sugar transporter subunit IIC
SAUSA300_RS02160	*ssl9*	Staphylococcal superantigen-like protein 9
SAUSA300_RS02600		Hypothetical protein
SAUSA300_RS02770		23S rRNA [guanosine(2251)-2'-*O*]-methyltransferase
SAUSA300_RS03050	*eutD*	Phosphate acetyltransferase
SAUSA300_RS03080		Dihydrolipoamide dehydrogenase
SAUSA300_RS03585		Hypothetical protein
SAUSA300_RS04015	*lgt*	Prolipoprotein diacylglyceryl transferase
SAUSA300_RS04025		Putative PEP-CTERM system TPR-repeat lipoprotein
SAUSA300_RS04580		Pyridine nucleotide-disulfide oxidoreductase
SAUSA300_RS05430	*typA*	Translational GTPase
SAUSA300_RS05545	*isdC*	Iron-regulated surface determinant protein C
SAUSA300_RS05555	*isdE*	Heme uptake system protein
SAUSA300_RS06485	*glnA*	Glutamine synthetase
SAUSA300_RS07355	*bshA*	*N*-Acetyl-alpha-d-glucosaminyl l-malate synthase
SAUSA300_RS07900	*xerD*	Tyrosine recombinase
SAUSA300_RS07905	*fur*	Ferric uptake regulator
SAUSA300_RS09130	*ptaA*	PTS glucose transporter subunit IIBC
SAUSA300_RS11445	*fbaA*	Fructose-bisphosphate aldolase
SAUSA300_RS11760	*htsA*	ABC transporter substrate-binding protein
SAUSA300_RS12210		AcrB/AcrD/AcrF family protein
SAUSA300_RS12780	*lctP2*	l-Lactate permease
SAUSA300_RS13075	*hlgC*	Gamma-hemolysin component C
SAUSA300_RS13330	*cntF*	ABC transporter ATP-binding protein
SAUSA300_RS13355	*cntM*	Staphylopine synthase
SAUSA300_RS13745	*cidC*	Pyruvate oxidase
SAUSA300_RS14605		DNA-binding protein

a Abbreviations: PTS, phosphotransferase system; PEP, proline-glutamate-proline; CTERM, C terminal; TPR, tetratricopeptide repeats.

Our results also revealed that our current *in vitro* models underrepresent the level of virulence factor expression that occurs during human infection, with many virulence factors showing significantly higher expression *in vivo* than *in vitro* (Data Set S1). Many of these virulence factors are directly regulated by the SaeRS two-component system regulon (16, 47), which is known to be responsive to host stimuli such as neutrophils and alpha-defensin (16). It is likely that the reduced expression of these factors *in vitro* is therefore due to the absence of an environmental cue. Importantly, we were able to increase the expression of virulence factors in the model system SCFM2 with the addition of an alpha-defensin (HNP-1), improving this model (Fig. 4). Although this led to only a modest improvement in the model, it is important as it indicates that one can target and improve key aspects of *in vitro* models to better mimic infection using transcriptomics. We focused on improving SCFM2 as it is a model that has been specifically developed to mimic the CF sputum environment. In addition to the supplementation of host molecules, another way one could potentially improve this model is with the use of CF clinical isolates instead of the skin and tissue isolates that comprised the majority of our *in vitro* samples. Finally, our data indicate that S. aureus is in a fermentative state under many of the *in vitro* conditions used. S. aureus requires vigorous shaking during *in vitro* culture to prevent it from entering fermentation; therefore, another possible way to improve these *in vitro* models is by increasing the aeration, preventing this switch to fermentation. Together, this work highlights the importance of choosing conditions that most closely mimic the aspects of the infection environment to be studied and indicate key areas in which models can be improved.

S. aureus has been studied for over >135 years (2), and in that time researchers have developed *in vitro* models with the goal of understanding S. aureus physiology within infection. While these models have been invaluable, we still do not fully understand how well and in what ways these models mimic the human infection environment. This study contributes to this long line of work as the largest assessment of global S. aureus transcription in human infection. Future research will build on these results and assess the ability of polymicrobial or spatially structured systems to better mimic the *in vivo* infection environment.

## MATERIALS AND METHODS

### Strains and culture conditions.

The USA300 community-associated methicillin-resistant Staphylococcus aureus strain LAC* (33) was used in this study. Isolates were routinely grown on brain heart infusion agar incubated at 37°C in ambient air. Chemically defined medium (CDM) was prepared as previously described (52) with the following modification for S. aureus: the amino acid and nucleotide stock solution was added at 0.25× to allow the primary carbon source to be exchanged as S. aureus can grow on amino acids. S. aureus was grown in SCFM2 as previously described (24). Briefly, 750 μl of SCFM2 in four-well microchamber slides from Nunc was inoculated at an optical density at 600 nm (OD_600_) of 0.05 of S. aureus strain LAC*, grown for either 3 or 7 h, and then immediately added to 5 volumes of RNAlater (ThermoFisher). Two technical replicates were combined for each biological replicate. For the addition of HNP-1 to SCFM2, 2.4 μM HNP-1 (Sigma-Aldrich) was added to SCFM2 at the time of inoculation and S. aureus was grown in four-well microchambers as described above. After 3 h of growth, samples were immediately added to 5 volumes of RNAlater. Planktonic cultures were grown at 37°C with shaking at 225 rpm and a flask-to-volume ratio of 5:1.

### RNA extraction and preparation of sequencing libraries for RNA-seq.

*In vitro* and human samples were prepared as previously described (24) with a few modifications for the human samples. For the human sputum samples, expectorated sputum was collected from adult patients who were clinically stable, immediately added to RNAlater, and stored at 4°C overnight and then at −80°C. Samples in RNAlater were thawed on ice and centrifuged at 4°C for 30 min at 10,000 × *g*. RNAlater was removed from the sample, and sputum was transferred to bead-beating tubes containing a mixture of large and small beads (2-mm zirconia and 0.1-mm zirconia-silica, respectively). *In vitro* cultures stored in RNAlater were pelleted, resuspended in 1 ml RNA-Bee, and transferred to bead-beating tubes. Samples were resuspended in RNase- and DNase-free TE buffer (Acros Organics), and lysozyme (1-mg/ml final concentration) and lysostaphin (0.17-mg/ml final concentration) were added to each sample. Samples were incubated at 37°C for 30 min to enzymatically lyse cells. RNA-Bee was added to each sample, and samples were lysed mechanically by bead beating three times for 30 s, placing the tubes on ice for ≥1 min between each homogenization. Amounts of 200 μl of chloroform per 1 ml of RNA-Bee were added, and the tubes were shaken vigorously for 30 s and incubated on ice for 5 min or overnight to allow phases to partition. Samples were centrifuged at 12,000 × *g* for 15 min at 4°C to separate the aqueous and organic phases. The aqueous phase from each tube was transferred to a new microcentrifuge tube to which 0.5 ml isopropanol per 1 ml of RNA-Bee was added in addition to 20 μg of linear acrylamide, and the tubes were incubated at −80°C overnight. Samples were thawed on ice and centrifuged at 12,000 × *g* for 30 min at 4°C. Pellets were washed with 1 ml 75% ethanol, air dried for 5 min, and resuspended in 100 μl of RNase-free water. The RNA concentration for each sample was determined with a NanoDrop spectrophotometer (Thermo Fisher Scientific). rRNA was depleted using the RiboZero Gold bacterial kit (Illumina) for the *in vitro* samples and the RiboZero Gold epidemiology kit (Illumina) for the human samples and purified by ethanol precipitation using linear acrylamide to help precipitate the RNA. The depleted RNA was fragmented for 2 min with the NEBNext Magnesium RNA fragmentation module kit, and cDNA libraries were prepared using the NEBNext Multiplex small RNA library prep kit (New England Biolabs) per the manufacturer’s instructions. Libraries were sequenced at the Molecular Evolution Core at the Georgia Institute of Technology by Illumina NextSeq500 75-bp single-end runs.

### Bioinformatic analysis of RNA-seq data.

RNA-seq reads were trimmed using Cutadapt 1.18 with a minimum read length threshold of 18 bases (53). Reads were mapped to a pangenome and collapsed onto orthologs in the S. aureus reference strain genome USA300_FPR3757 (accession number GCF_000013465.1) downloaded from the National Center for Biotechnology Information using Bowtie 2.3.2 with the default parameters for end-to-end alignment (54). Reads were tallied and assigned to only those genes common to all strains in the S. aureus 15-strain pangenome (1,960 genes [see Table S1 in the supplemental material) using the *htseq-count* function in the HTSeq package (55) (v. 0.11.2-0). Reads mapping to rRNAs and tRNAs were removed from the analysis. The remaining raw reads were normalized using the *estimateSizeFactors()* function and transformed using the *varianceStabilizingTransformation()* function in the DESeq2 package prior to analysis. Principal components were determined using the *prcomp()* function. In addition to this methodology, we also performed this analysis using a subset of genes where all human samples had at least 1 read in all of the genes used, leading to a common set of 1,046 genes. Principal components were determined in the same way as described above. Differential expression was determined with DESeq2 (37) with betaPrior set to true. The hierarchal clustering was performed in the heatmap3 R package (56). Heatmaps were generated from varianceStabilizingTransformation normalized read counts using Prism 7 (GraphPad). For the machine learning component of the paper, the R package mlr was used (57). Feature selection and validation were performed using a wrapper and the “*FSelectorRccp_information.gain*” filter to select 50 features, and this was combined with 10-fold cross-validation and leave-one-out cross-validation methods. We chose 32 features that were used in all iterations of cross-validation to build and train the SVM model.

### Ethical statement.

Expectorated CF sputum samples for this study were collected from Emory + Children’s Center for Cystic Fibrosis and Airways Disease Research as previously described by our group (24) with IRB approval (Georgia Tech approval no. H18220).

### Data availability.

The raw sequencing files from this study are available at the NCBI Sequence Read Archive (SRA) under accession number SRP222773. The accession numbers for previously published samples used are listed here: *in vitro* (SRP178123, SRP048673, and SRP066096) and human (SRP048673 and SRP135669).
