# Supplementary material for: The Staphylococcus aureus Transcriptome during Cystic Fibrosis Lung Infection
Source: mBio. 2019 Nov 19;10(6):e02774-19. doi: 10.1128/mBio.02774-19 (PMC6867902; doi:10.1128/mBio.02774-19)
Supplement: TABLE S1 [file mBio.02774-19-st001.docx]

| Strain Name | Accession Number | Sequence length | Number of Protein Coding Genes | Clonal Complex/Sequence Type |
| --- | --- | --- | --- | --- |
| N315 | GCF_000009645.1 | 2,814,816 | 2776 | CC5 |
| USA300_FPR3757 | GCF_000013465.1 | 2,872,769 | 2917 | CC8 |
| 04-02981 | GCF_000025145.1 | 2,821,452 | 2776 | CC5 |
| ED98 | GCF_000024585.1 | 2,824,404 | 2794 | CC5 |
| JH1 | GCF_000017125.1 | 2,906,507 | 2934 | CC5 |
| JKD6008 | GCF_000145595.1 | 2,924,344 | 2903 | ST239 |
| LGA251 | GCF_000237265.1 | 2,750,834 | 2632 | CC130 |
| MRSA252 | GCF_000011505.1 | 2,902,619 | 2819 | CC30 |
| MSSA476 | GCF_000011525.1 | 2,799,802 | 2770 | CC1 |
| MW2 | GCF_000011265.1 | 2,820,462 | 2778 | CC1 |
| ST398 | GCF_000009585.1 | 2,872,582 | 2783 | CC398 |
| TCH60 | GCF_000159535.2 | 2,802,675 | 2691 | CC30 |
| TW20 | GCF_000027045.1 | 3,043,210 | 3075 | ST239 |
| USA300_TCH1516 | GCF_000017085.1 | 2,872,915 | 2900 | CC8 |
| VC40 | GCF_000245495.1 | 2,692,570 | 2640 | CC8 |
|  |  | Average | Average |  |
|  |  | 2,846,360 | 2813 |  |

Table S1. S. aureus strains used to construct the reduced gene set of 1960 conserved genes
