# Supplementary material for: The Staphylococcus aureus Transcriptome during Cystic Fibrosis Lung Infection
Source: mBio. 2019 Nov 19;10(6):e02774-19. doi: 10.1128/mBio.02774-19 (PMC6867902; doi:10.1128/mBio.02774-19)
Supplement: TABLE S2 [file mBio.02774-19-st002.docx]

Supplementary Table 2. Assessment of SVM model performance after removing co-regulated genes from the 32 gene transcriptional signature of human CF infection.^1^

| Condition | Probability Human |
| --- | --- |
| SCFM2 mid-exponential | 0.057±0.016 |
| SCFM2 late-exponential | 0.157±0.089 |
| Danish human sputum | 0.783 |
| Human chronic wound | 0.342 |
| Human joint infection | 0.373 |

^1^Genes used for this analysis (28 genes): *SAUSA300_RS00540*, *sbnI*, *uhpT*, *SAUSA300_RS01655*, *ssl9*, *SAUSA300_RS02600*, *SAUSA300_RS02770*, *eutD*, *SAUSA300_RS03080*, *SAUSA300_RS03585*, *lgt*, *SAUSA300_RS04025*, *SAUSA300_RS04580*, typA, *isdE*, *glnA*, *bshA*, *xerD*, *ptaA*, *fbaA*, *htsA*, *SAUSA300_RS12210*, *lctP2*, *hlgC*, *cntF*, *cidC*, and *SAUSA300_RS14605*. We found 100% accuracy with both k-fold (10 iterations) and leave-one-out cross validation of the training dataset with this modified transcriptomic signature.
